# More comprehensive sex education reduced teen births: Quasi-experimental evidence

**DOI:** 10.1073/pnas.2113144119

**Published:** 2022-02-14

**Authors:** Nicholas D. E. Mark, Lawrence L. Wu

**Affiliations:** ^a^Department of Sociology, New York University, New York, NY 10012

**Keywords:** teen births, sex education, demography

## Abstract

Women in the United States are much more likely to become mothers as teens than those in other rich countries. Teen births are particularly likely to be reported as unintended, leading to debate over whether better information on sex and contraception might lead to reductions in teen births. We contribute to this debate by providing causal evidence at the population level. Our causal identification strategy exploits county-level variation in the timing and receipt of federal funding for more comprehensive sex education and data on age-specific teen birth rates at the county level constructed from birth certificate natality data covering all births in the United States. Our results show that federal funding for more comprehensive sex education reduced county-level teen birth rates by more than 3%. Our findings thus complement the mixed evidence to date from randomized control trials on teen pregnancies and births by providing population-level causal evidence that federal funding for more comprehensive sex education led to reductions in teen births.

## Significance

Sex education for youth in the United States has been the topic of considerable debate among researchers, policy makers, and the public at large. In this study, we focus attention on federal funding for more comprehensive sex education that was received by a mix of public and private organizations in 55 US counties. Our analyses provide population-level causal evidence that funding for more comprehensive sex education led to an overall reduction in the teen birth rate at the county level of more than 3%. This study thus contributes causal evidence relevant to ongoing debates on the potential role more comprehensive sex education may play in reducing teen births in the United States.

The United States has one of the highest teen birth rates among rich countries (1), a distinction that has long sustained the interest of academics, politicians, and the public (2–4). Teen births are also much more likely to be reported as unintended than births at older ages (5). The federal government has responded, in part, by funding two types of sex education for America’s teens: abstinence-only sex education that promotes abstaining from sex until marriage and more comprehensive sex education that includes scientifically and medically accurate information about contraception and reproductive health.

A broad research base has shown that abstinence-only programs are ineffective at reducing teen birth rates (6–13). Evaluations of randomized control trials (RCTs) of teen pregnancy prevention programs providing more comprehensive sex education have yielded mixed findings, with inconclusive results for teen pregnancies and births, but more positive results for outcomes such as knowledge about contraception and sexual and reproductive health and the development of skills associated with healthy relationships (14, 15). These mixed findings have led at least some to question whether the widespread adoption of such programs would, in fact, alter the behavior of sexually active teens (15, 16).

We provide causal evidence at the population level that federal funding for more comprehensive sex education led to reductions in teen births. Our causal identification strategy exploits variation in the timing and receipt of county-level funding. We compare age-specific teen birth rates for counties in the same state and year that did and did not receive federal funding using difference-in-differences (DiD) specifications with county and state-year fixed effects. We find that federal funding for more comprehensive sex education led to an overall reduction of more than 3% in the rate of teen births at the county level.

## Background

Federal funding directly supporting efforts by local organizations to teach sex education has alternated between funding for abstinence-only and more comprehensive programs. In the 1990s, funding for abstinence-only programs was predicated on a strict eight-point definition of abstinence-only sex education outlined in the Personal Responsibility and Work Opportunity Reconciliation Act (PRWORA) of 1996.* These criteria require providers to avoid the topic of contraception except to emphasize contraceptive failure rates.

Federal funding for teen pregnancy prevention programs providing more comprehensive sex education began in 2010 with two programs: the Personal Responsibility Education Program (PREP), which provided funding to states, and the Teen Pregnancy Prevention program (TPP), which provided funding at the county level.^†^ TPP awarded funds competitively to local public and private entities to “replicate evidence-based teen pregnancy prevention program models that have been shown to be effective through rigorous evaluation research” and to “develop and test additional models and innovative strategies to prevent teen pregnancy” (17). Competitive grant funding under PREP was intended to help prevent teen pregnancy and the spread of sexually transmitted infections (STIs) by delaying sexual activity and increasing condom and contraceptive use. TPP and PREP funded programs for middle- and high school-aged students but allowed programming to be delivered in a variety of settings, including schools, community centers, and medical clinics. Both eliminated the requirement that programs adhere to the eight-point abstinence-only mandate of PWRORA-funded programs, although programs were allowed discretion over whether to include abstinence as one means of avoiding a pregnancy. The approach and the topics covered thus varied substantially across funded programs. Although many interventions funded by TPP did not, for example, meet the National Sex Education Standards published by Future of Sex Education Initiative (18), the vast majority provided more comprehensive information on sex, contraception, and reproductive health than abstinence-only programs.

Because our causal identification strategy relies on county-level variation in the timing and receipt of federal funding, we focus attention on TPP. TPP funding averaged more than $100 million per year and reached ∼980,000 teens between 2010 and 2016 (19, 20). Funds were awarded in two tiers, with Tier 1 funding evidence-based programs and Tier 2 funding tests of new programs. About three-quarters of the funding each year was for Tier 1 programs; the other one-quarter went to Tier 2. In 2010 at the outset of TPP, there were 28 programs that qualified for Tier 1 (17), but, by 2018, there were 44 (21).

Francis et al. (22) provide a description of the Teen Outreach Program (TOP), an existing evidence-based program typical of those receiving Tier 1 funding. TOP, which was successful when first implemented in 1997, was a “youth development” and “service-learning” program whose primary goal was to reduce adolescent pregnancies. It consisted of three components: 1) weekly curriculum sessions, 2) community service learning, and 3) positive adult guidance and support. Program facilitators selected lessons from the following topics based on the perceived needs of the youths: values clarification, relationships, communication/assertiveness, influence, goal setting, decision-making, and human development and sexuality. In 2014 alone, TOP was offered in 35 states, affecting 35,000 adolescents (22).

Other contemporary publications also shed some light on what implementation looked like on the ground, including what forms the programs took, where they were delivered, and which populations of teens they were delivered to. The Office of Adolescent Health contracted Abt Associates to evaluate the implementation and impacts of three evidence-based program models: Reducing the Risk (RtR), ¡Cuidate!, and Safer Sex Interventions (SSI). SSI, a clinic-based program focused on HIV/AIDS prevention, was implemented by clinic operators such as Planned Parenthood and county health departments. RtR, a curriculum-based program focused on sexual health and risk prevention, was implemented in classrooms during the school day. ¡Cuidate!, a curriculum-based program focused on HIV/STI risk reduction, was targeted specifically to Latino adolescents.

Organizations receiving TPP funding for these programs targeted different groups of at-risk youth. For example, RtR was delivered in San Diego to 9th and 10th grade students in the county in schools identified as “teen pregnancy hotspots” by the state, while, in St. Louis, RtR was delivered to a population that was “almost entirely” low-income and Black. In Knox County, TN, SSI was delivered by the county health department to “teen pregnancy hotspots” and “children in state custody” (23–27).

### Prior Evidence.

The observational and RCT evidence on the effects of more comprehensive sex education has been mixed (8, 15, 21, 28). A consistent finding from observational studies that rely on regression analyses of survey data is that comprehensive sex education is associated with lower pregnancy risks, a later age at first sex, and an increased probability of contraceptive use (29–31). By contrast, the evidence from RCT evaluations, including those of TPP, have been far more mixed. For example, a review of 19 replications of previously successful TPP programs found that about 20% replicated and 42% did not, with the balance inconclusive because of compromised design implementation (28). The same review analyzed data from 22 demonstrations of new programs implemented under TPP. Of these, 36% caused moderate declines in pregnancy rates and rates of sex without contraception, 36% had null effects, and 27% were compromised (28). One large TPP-funded program examined whether a previously successful TPP program remained effective when scaled up, but found no impact at scale (22). A metaanalysis of both TPP replications and new programs found that they had only small and statistically insignificant effects on behavioral outcomes such as ever having sex, having sex recently, and having sex without contraception (15). These failures of previously successful TPP interventions—to replicate, to demonstrate effectiveness in different subgroups, or to be effective when implemented at scale—have prompted some to question whether renewed funding for more comprehensive programs like TPP and PREP would, in fact, lead to changes in the behaviors of teens and young adults (16).

This contrast between RCT and observational evidence may reflect the strengths and weaknesses of their respective designs. Often touted as a “gold standard,” RCTs require substantial commitments of time and effort. Funding constraints thus often require researchers to restrict attention to particular subgroups and relatively short follow-up periods, thus limiting statistical power and the range of outcomes to be evaluated (32). This was true of the RCTs funded by TPP. Roughly one-third targeted middle school students only, and, although most examined contraceptive use ( ∼80%) and sexual activity ( ∼73%), far fewer examined pregnancies ( ∼20%), and none examined births given the typical 9- to 24-mo follow-up period (15, 33, 34). Finally, the local nature of any RCT raises two related but conceptually distinct questions: whether findings generalize at the population level and whether treatment can be effectively scaled up in settings where treatment may be implemented with less fidelity than the original RCT (34).

By contrast, the observational studies cited above relied on nationally representative survey data that included retrospective self-reports by female respondents on sex education and births, thus capturing long-term impacts at the population level. But these studies almost certainly overstate the true relationship between comprehensive sex education and teen births, due to their inability to control for all potentially important confounds and because of the likely nonrandom selectivity of those who recall enough about their sex education to categorize it as comprehensive.

Our analyses address key weaknesses in the survey and RCT studies reviewed above. In contrast to the correlational evidence from studies analyzing survey data, our quasi-experimental identification strategy uses exogenous variation in the timing and receipt of funding to obtain plausibly causal estimates of the effect of more comprehensive sex education on teen birth rates at the county level. And, in contrast to RCT evaluations, our use of natality data obtained from US birth certificates provides not only evidence at the population level but enough statistical power to obtain estimates with reasonable precision.^‡^

An important limitation of our study is the binary nature of our treatment variable—whether a county did or did not receive federal funding for more comprehensive sex education in a given calendar year. Thus, for a county receiving funding, we do not know which or how many teens were treated during the years in which funding was received. This, in turn, implies that we cannot identify effects at the individual level and, hence, that our results should instead be interpreted as the effect of funding on teen births at the county level. Nevertheless, decreases in teen births at the county level that are the causal consequence of treatment are possible if and only if there were, on average, decreases in teen births due to treatment at the individual level.

## Data and Methods

### Data.

#### Data on teen births.

To obtain teen birth rates at the county level, we merged data from two sources. County-level data on births were obtained from restricted natality data from the National Vital Statistics System (NVSS) (35).^§^ The NVSS natality data are derived from birth certificates and cover all births, in a given calendar year, to women in the United States. We use information on the mother’s age to the nearest year and the mother’s county of residence. We restrict our analysis to births to teens aged 14 y to 19 y, excluding births at younger ages because very few births at age 13 y or younger were recorded.

We obtained annual estimates of county-level populations by single years of age from the Survey of Epidemiology and End Results (SEER) conducted by the National Cancer Institute (36). The birth rate at age *x* in county *i* in calendar year *t* is then given by the ratio of births at age *x* in county *i* and calendar year *t* (numerators from NVSS) to the number of women who, at age *x*, resided in county *i* in calendar year *t* (denominators from SEER).

#### Data on federal funding for more comprehensive sex education.

Data on grants from federally funded sex education programs are publicly available from the System for Award Management’s Catalog of Federal Domestic Assistance (CFDA) (37), which we used to identify counties that received TPP funding for more comprehensive sex education. The data for each award contain a unique CFDA identifier and the recipient’s name, address, and dates of project performance.

As noted previously, our analyses employ simple county-level binary indicators for receipt of funding in a given calendar year because the funding data lack more detailed information, for example, on the numbers, ages, or types of students in a given county and calendar year who received instruction on sex education from a funded program.^¶^

#### Time-varying covariates.

Most county-level factors change little over time and will thus be accounted for by the county fixed effects. However, fertility may be influenced by local economic conditions in ways that may vary with age (38, 39). Given that funding for more comprehensive sex education became available shortly after the official end of the Great Recession, we adjust for annual county unemployment using local area unemployment rates from the Bureau of Labor Statistics (40), as well as annual median household income and poverty rates from the Census Bureau’s Small Area Income and Poverty Estimates program (41). Our models also adjust for the age and racial demographics of the county’s teens using the data from SEER. These variables are the percent of teens that are ages 16 y to 19 y and the percent of teens that are White, Black, and Hispanic.

Finally, our models adjust for changes in abortion availability using a measure of the distance to the nearest abortion provider. This county-level measure is available for the calendar years 2000, 2011, and 2014 and gives the median distance to the nearest abortion provider weighted by the number of females in the county aged of 15 y to 44 y (42). We used linear interpolation to estimate values of this measure for the years between 2000 and 2014, and linear extrapolation to obtain values before 2000 and after 2014.

#### Sample criteria.

Our analytic sample consists of county-year observations for the period 1996–2017. We dropped a county 1) if the number of females in the county aged 14–19 y fell to zero or if its reporting area changed between 1996 and 2017 (*n* = 21); 2) if the county had previously received federal funding for abstinence-only sex education (*n* = 173) under the Community-Based Abstinence Education program (43); 3) if the county was missing at least one year of data on any of the time-varying covariates listed above (*n* = 24); or 4) if the county’s funding was not included in the two main cohorts of awards (*n* = 4). Counties in Hawaii were also excluded because natality data in Hawaii were not disaggregated by county until 2000. These exclusions reduced the number of counties in our analytic sample to 2,927. By excluding counties that had previously received abstinence-only funding, the resulting control and treatment conditions consist of county-years in a never-funded condition (control condition) vs. county-years in which the first and only source of county-level federal funding was for more comprehensive sex education (treatment condition). Our results are, however, generally robust to the inclusion or exclusion of the 173 counties that received abstinence-only funding (*SI Appendix*, Table S1).

**Fig. 1.**
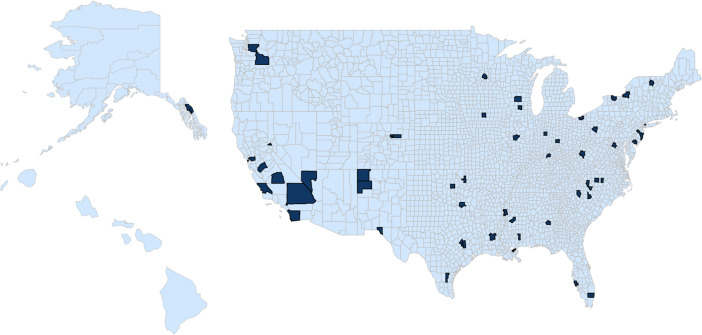
Map of counties in the sample that received funding for more comprehensive sex education.

Fig. 1 displays a map of counties in the United States, highlighting those that exclusively received county-level federal funding for more comprehensive sex education and are thus part of our analytic sample. Funded counties were geographically dispersed, showing that funding was not concentrated in any particular state or region.

Table 1 reports the funding status of counties for the period 1996–2016.^#^ As noted above, funding for more comprehensive sex education began in 2010; hence, the first row of Table 1 refers to the pretreatment period for the years 1996–2009. Of the 2,927 counties in our analytic sample, 55 were home to organizations that received federal funding for more comprehensive sex education. The number of counties receiving funding rose from 36 in 2010 to 55 in 2016.

**Table 1.** Counties by funding status

	Funding status		
Year	Not funded	Funded	Total
1996–2009	2,927	0	2,927
2010	2,891	36	2,927
2011	2,891	36	2,927
2012	2,891	36	2,927
2013	2,891	36	2,927
2014	2,891	36	2,927
2015	2,872	55	2,927
2016	2,872	55	2,927

### Methods.

To obtain plausibly causal estimates of the effect of federal funding for more comprehensive sex education on teen birth rates at the county level, we use DiD procedures to exploit plausibly exogenous variation.

Model 1 specifies county and state-year fixed effects that we use throughout to compare counties in the same state and year that did and did not receive federal funding for more comprehensive sex education,

[1] $$\log\;[1+R_{\mathrm{ijt}}(x)]=\beta_{0}+\beta_{1}f_{\mathrm{ijt}-1}+\theta_{i}+\phi_{jt}+\epsilon_{\mathrm{ijt}},$$

where $R_{\mathrm{ijt}}(x)$ denotes the number of births at age *x* per 1,000 women who were age *x* in county *i*, state *j*, and year *t*; $f_{\mathrm{ijt}-1}$ is our binary treatment variable, which is lagged by 1 y to account for the time for a conception to be taken to term; *θ_i_* and $\phi_{jt}$ are the county and state-year fixed effects; and *ϵ_ijt_* is a normally distributed error term clustered at the county level. In model 1, we have also taken the log of the quantity $1+R_{\mathrm{ijt}}(x)$ to allow *β*_1_ to be roughly interpretable as the percent change in *R* due to funding for more comprehensive sex education. Note that the state-year fixed effect $\phi_{jt}$ differs from separate state and year fixed effects, with $\phi_{jt}$ thus adjusting, for example, for a state-level sex education mandate affecting all counties in the state during the calendar years in which the mandate was in effect.

Model 2 differs from model 1 by including county-level measures of $d_{\mathrm{ijt}-1}$, the distance to an abortion provider; $U_{\mathrm{ijt}-1}$, a vector for the unemployment rate, median household income, and the poverty rate; $a_{\mathrm{ijt}-1}$, the percent of teen women who are 16 y to 19 y old; and $D_{\mathrm{ijt}-1}$, a vector for the percent of teen women who are White, Black, and Hispanic. Each of these covariates is lagged 1 y.

[2] $$\begin{array}{l} \log\;[1+R_{\mathrm{ijt}}(x)]=\gamma_{0}+\gamma_{1}f_{\mathrm{ijt}-1}+\gamma_{2}d_{\mathrm{ijt}-1}+\gamma_{3}U_{\mathrm{ijt}-1}+\gamma_{4}a_{\mathrm{ijt}-1}\\ \;+\gamma_{5}D_{\mathrm{ijt}-1}+\theta_{i}+\phi_{jt}+\epsilon_{\mathrm{ijt}}. \end{array}$$

Model 3 adds county-specific linear time trends, $\theta_{i}*T$, to model 2. This term adjusts for heterogeneous trends in teen birth rates at the county level.

[3] $$\begin{array}{l} \log\;[1+R_{\mathrm{ijt}}(x)]=\alpha_{0}+\alpha_{1}f_{\mathrm{ijt}-1}+\alpha_{2}d_{\mathrm{ijt}-1}+\alpha_{3}U_{\mathrm{ijt}-1}+\alpha_{4}a_{\mathrm{ijt}-1}\\ \;+\alpha_{5}D_{\mathrm{ijt}-1}+\theta_{i}+\theta_{i}*T+\phi_{jt}+\epsilon_{\mathrm{ijt}}. \end{array}$$

**Fig. 2.**
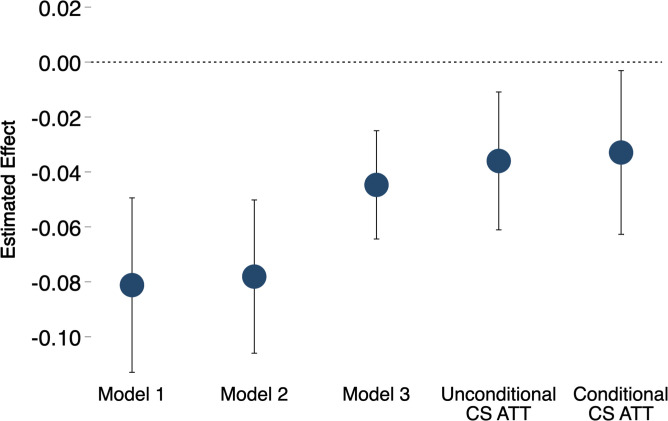
Alternative estimates of the effect of federal funding for more comprehensive sex education on the county-level teen birth rate. Teens are aged 14 y to 19 y.

**Fig. 3.**
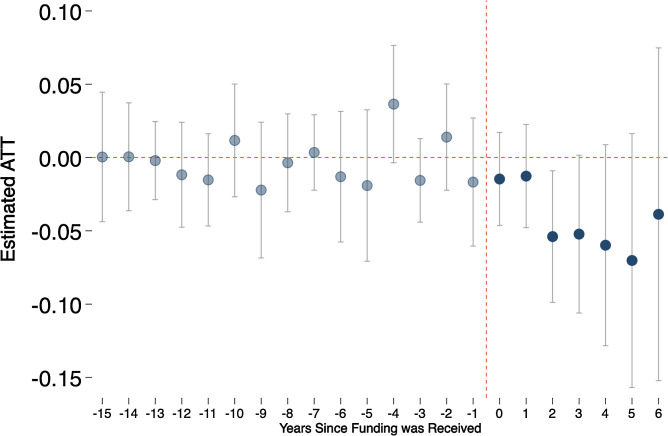
Estimated causal effect of funding for more comprehensive sex education by year prior to or following funding receipt.

Although many studies using DiD methods have interpreted the regression coefficients *β*_1_, *γ*_1_, or *α*_1_ as the average effect of treatment on the treated (ATT), recent work has shown that these regression coefficients are not, in fact, ATTs; see, e.g., Goodman-Bacon (44), de Chaisemartin and D’Haultfœuille (45), and Callaway and Sant’Anna (46). To obtain estimates of the ATT and other quantities reported below in Figs. 2–3, we have used estimation procedures proposed by ref. 46 (hereafter, the procedures are denoted as CS), who show that these estimation procedures are asymptotically consistent when treatment adoption is staggered at different times, as is the case here with different counties receiving funding in different years. We report both unconditional and conditional versions of the CS ATT, with the conditional CS ATT using the covariates in model 2 to match treatment and controls in the pretreatment period.^‖^

These procedures also permit estimates of effects by time since treatment. In the pretreatment period, these estimates facilitate judgments about the plausibility of the parallel trends assumption inherent in DiD designs.** In the posttreatment period, they illustrate the treatment effects by time since treatment.

## Results

Fig. 2 presents estimates of the effect of federal funding for more comprehensive sex education on county-level teen birth rates at ages 14 y to 19 y.

We have plotted five alternative estimates: the treatment coefficients from the models 1, 2, and 3 regressions and the conditional and unconditional CS ATT. The model 1 estimate suggests that federal funding resulted in a statistically significant reduction of about 8.1% (95% CI = –11.3%, –4.9%) in county-level birth rates. The model 2 estimate, which adjusts for time-varying covariates, is slightly larger at –8.6% (95% CI = –11.3%, –5.9%). The model 3 estimate, adjusting for county-specific trends, is smaller, at –4.4% (95% CI = –6.3%, –2.5%). The unconditional CS ATT is still smaller, at –3.6% (95% CI = –6.1%, –1.1%), and the conditional CS ATT, our preferred estimate, is –3.3% (95% CI = –6.3%, –0.3%). To put this 3.3% reduction in context, it is about half the size of the estimated effect on teen births of the 2009 Colorado Family Initiative, a program that expanded contraceptive access in Colorado via Title X clinics (48).

Fig. 3 provides estimates of the effects of funding in the pretreatment and posttreatment periods for the conditional ATTs. In the pretreatment period, Fig. 3 shows the year-on-year change for the treatment compared to control groups. The pretreatment estimates fluctuate around zero, are not statistically significant, and pass a Wald test of a difference in pretreatment trends for the treatment and control group, thus providing evidence that the parallel trend assumption holds in the pretreatment period. That the parallel trend assumption holds in the pretreatment period strengthens the case that this assumption will hold in the posttreatment period—that trends would be parallel under the counterfactual in which treatment were, in fact, not to occur for treated counties.

Estimates in the posttreatment period increase from about –1.5% (95% CI = –4.6%, 1.7%) in the first year of funding to approximately – 7.0% (95% CI = –15.7%, 1.6%) in the fifth year of funding. This posttreatment pattern, in which estimates become more negative with time since treatment, is consistent with the RCT findings of small, nonsignificant impacts on pregnancies and other behavioral outcomes in the immediate posttreatment period. But they suggest that federal funding did have effects in later years, which may be due to increases over time in the numbers of teens who were treated, or due to teens who received programming at earlier ages aging into years in which they became more sexually active.

*SI Appendix* reports a series of additional analyses showing that our findings appear robust to a variety of alternatives concerning model specification, weighting, and the sampling of counties.

## Discussion

The findings in this paper provide quasi-experimental evidence on the causal effect of federal funding for more comprehensive sex education on teen births. We find that federal funding reduced the overall rate of teen births at the county level by more than 3%. These results thus complement the mixed findings from RCTs by providing population-level evidence on the causal role played by more comprehensive sex education.

It is likely that our findings understate the true effect of more comprehensive sex education at the individual level. On the one hand, our quasi-experimental evidence shows that the federal funding received by local organizations played a causal role in reducing teen births at the county level. On the other hand, our binary funding indicator for whether any organization in the county received federal funding ignores other critical aspects such as the numbers of teens treated, the specific topics covered, or the fact that some funded programs, in fact, provided little or no comprehensive information on ways to prevent a pregnancy. It is thus only a limited proxy for whether or not an individual teen received more comprehensive sex education. On balance, these and other factors could imply that our causal evidence is conservative with respect to the magnitude of the true effect of federal funding for more comprehensive sex education on individuals.

Our findings leave many questions unanswered. First, our focus on teen births examines only one aspect of the multifaceted nature of sex education, thus ignoring whether more comprehensive sex education might affect other sexual, reproductive, or developmental outcomes (18). Reductions in teen births are thus only one way in which more comprehensive sex education may influence adolescent and young adult behaviors.

Second, our findings do not speak to the specific mechanisms by which more comprehensive sex education resulted in fewer teen births. One possibility is suggested by a consistent finding from RCT evaluations of teen pregnancy prevention programs—that they had positive effects on attitudes and knowledge about contraception, pregnancy prevention, and overall sexual health for treated teens relative to controls (28). Our findings on teen births, most of which will have occurred well after the typical RCT had ended, may thus point to the causal role played by changes in attitudes and knowledge on the subsequent risk of a teen pregnancy or birth.

Third, our findings speak only to the actual mix of programs implemented by funded counties, leaving open the question of whether they generalize to a different mix of programs. Our quasi-experimental design also provides estimates only of the effect of treatment on the treated, leaving unanswered the question of whether effects would be similar for untreated counties that did not receive funding. Still, more comprehensive sex education could, in principle, be implemented using standardized curricula, raising the possibility that the reductions in teen births caused by funding for more comprehensive sex education might also hold at scale for the 2,800+ counties that did not receive funding. That unfunded counties saw fewer reductions in teen births thus could reflect an unmet need for effective ways to reduce teen pregnancies and births and, if so, that teens in counties that never received funding could benefit from more comprehensive sex education.

Finally, our results do little to explain the overall decline in teen births over the past three decades. The causes of this decline likely lie in the changing economic and societal context in which teen childbearing takes place.

## Supplementary Material

Supplementary File

## Data Availability

All publicly available data used in this paper are available in GitHub at https://github.com/NicholasDEMark/sex_ed. The publicly available data do not include the National Center for Health Statistics (NCHS) Natality files or the data on distance to an abortion provider. These two datasets are governed by confidentiality agreements with NCHS and The Guttmacher Institute, respectively. Researchers interested in replicating this paper’s results are encouraged to contact N.D.E.M. (nm2648@nyu.edu) for guidance on obtaining and using these data.

## Footnotes

*To receive abstinence-only funding, PRWORA required an instructional program that 1) has as its exclusive purpose teaching the social, psychological, and health gains to be realized by abstaining from sexual activity; 2) teaches abstinence from sexual activity outside marriage as the expected standard for all school-age children; 3) teaches that abstinence from sexual activity is the only certain way to avoid out-of-wedlock pregnancy, sexually transmitted diseases, and other associated health problems; 4) teaches that a mutually faithful monogamous relationship in the context of marriage is the expected standard of sexual activity; 5) teaches that sexual activity outside of the context of marriage is likely to have harmful psychological and physical effects; 6) teaches that bearing children out of wedlock is likely to have harmful consequences for the child, the child’s parents, and society; 7) teaches young people how to reject sexual advances and how alcohol and drug use increase vulnerability to sexual advances; and 8) teaches the importance of attaining self-sufficiency before engaging in sexual activity.

^†^We use the term “county-level” to refer to funds that were distributed to organizations in a county, even though such organizations may not have operated county-wide or exclusively in a single county.

^‡^Recent work has begun to use quasi-experimental designs similar to ours. Fox et al. (12) conducted a DiD analysis of funding for both abstinence-only and more comprehensive sex education using states as the unit of analysis and found no effect of either on teen births, except in more-conservative states. However, the majority of federal funding for more comprehensive sex education was distributed to entities at more-local levels and not to states. This introduces considerable measurement error in their state-level treatment variable, likely biasing their estimates toward zero. In *SI Appendix*, we present state-level analyses and show that adjusting for pretreatment demographics or state-specific trends in teen births results in estimated null effects of funding. This result implies that specifying treatment at the state level does indeed appear to bias estimates toward zero.

^§^Access to, and use of, restricted versions of national vital statistical files require research proposal review and approval by the NCHS. Researchers can request access to restricted-use files from NCHS through the process outlined on the restricted-use vital statistics data page or request access to the data through the NCHS Research Data Center.

^¶^While many recipients operated at smaller geographic levels, some funding was awarded to entities that may have provided programming in multiple counties. As our analyses compare counties that did and did not receive funding in the same state and year, this would bias the estimates toward zero.

^#^As we note below, we lag funding by 1 y to account for the time for a conception to be taken to term. For this reason, the funding data in Table 1 cover the period 1996–2016 while the data on teen births cover the period 1996–2017, with the 1-y lag assuming, for example, that funding in 2016 will affect births in 2017.

^‖^We used the R package, did (version 2.0), to obtain the CS quantities reported in Figs. 2–3. For these quantities, we defined the outcome for county *i* in state *j* and year *t* as the log of one plus the difference between the birth rate in county *i* and year *t* and the average birth rate for all counties in state *j* and year *t*. For the conditional CS ATT, the did package implements the matching procedures in Heckman et al. (47), which we used to match treatment and controls on the model 2 covariates set at their 2010 values.

**Some remaining issues could bias or pose threats to a causal interpretation of our CS estimates. A first is that federal funding likely did not treat all teens in funded counties or, relatedly, that different levels of funding would lead to different numbers of teens who were treated. Note, however, that this would typically bias estimates from Eqs. **1**–**3** toward zero, resulting in conservative estimates of the causal effect of funding. A second is that funding may have led teens or their families to move from one county to another, with mobility of concern only if the factors prompting mobility were correlated with treatment, which is unlikely. A third issue is the possibility that federal funding led to the reallocation of state funds from untreated to treated counties. It seems far more likely that any reallocation of state funds would involve flows from treated to untreated counties (biasing estimates toward zero) rather than from untreated to treated counties, but the latter would pose a threat to the causal interpretation of estimates we report.
