# Supplementary material for: More comprehensive sex education reduced teen births: Quasi-experimental evidence
Source: Proc Natl Acad Sci U S A. 2022 Feb 14;119(8):e2113144119. doi: 10.1073/pnas.2113144119 (PMC8872707; doi:10.1073/pnas.2113144119)
Supplement: Supplementary File [file pnas.2113144119.sapp.pdf]

Supporting Information for: More Comprehensive Sex Education Reduced Teen Births: Quasi-Experimental Evidence

Nicholas D.E. Mark and Lawrence L. Wu

January 4, 2022

1 County characteristics by funding status.

Table S1 presents descriptive statistics for treatment and control counties for the analytic sample, the sample in which rural counties are dropped, and the sample consisting of counties that received federal funding for either abstinence-only or more comprehensive sex education. Data are from NCHS natality files, SEER, SAIPE, BLS, and The Guttmacher Institute.

Table S1: County-level Descriptive Statistics

	Analytic Sample		Rural Counties Excluded		Only Federally Funded Counties	
Variable	Control	Treatment	Control	Treatment	Control	Treatment
Birth Rate per 1000	30.31	31.49	27.43	31.18	31.54	31.45
	(17.02)	(15.50)	(15.30)	(15.26)	(15.73)	(15.62)
Population of Teens 14-19	12,400	48,753	16,474	49,638	36,812	48,552
	(14,796)	(34,519)	(15,638)	(34,260)	(29,526)	(35,684)
Unemployment Rate	5.156	5.161	5.163	5.162	5.158	5.160
	(0.438)	(0.438)	(0.438)	(0.438)	(0.438)	(0.438)
Median Distance to an	40.22	12.00	25.00	10.37	16.49	12.63
Abortion Provider	(49.68)	(26.50)	(36.08)	(15.30)	(32.10)	(27.94)
Percent of Households	13.07	14.76	11.87	14.64	14.28	15.11
in Poverty	(5.848)	(6.058)	(5.297)	(5.898)	(5.575)	(6.249)
Median Household	48,185	48,409	52,342	48,635	48,418	48,059
Income	(15,491)	(13,655)	(15,559)	(13,581)	(14,452)	(14,134)
Percent of Teens	16.61	16.62	16.65	16.61	16.71	16.58
Aged 14	(2.382)	(1.666)	(2.237)	(1.658)	(1.351)	(1.693)
Percent of Teens	16.72	16.68	16.71	16.67	16.81	16.65
Aged 15	(2.341)	(1.585)	(2.183)	(1.575)	(1.256)	(1.612)
Percent of Teens	16.74	16.64	16.69	16.62	16.80	16.62
Aged 16	(2.287)	(1.508)	(2.112)	(1.498)	(1.187)	(1.537)
Percent of Teens	16.68	16.54	16.60	16.53	16.71	16.54
Aged 17	(2.196)	(1.411)	(2.007)	(1.400)	(1.137)	(1.440)
Percent of Teens	16.62	16.64	16.62	16.65	16.49	16.67
Aged 18	(2.393)	(1.624)	(2.276)	(1.610)	(1.376)	(1.633)
Percent of Teens	16.64	16.88	16.74	16.92	16.49	16.94
Aged 19	(6.436)	(4.412)	(6.089)	(4.399)	(3.326)	(4.522)
Percent of Teens	71.82	49.98	69.36	50.10	54.58	49.10
who are White	(21.15)	(22.27)	(20.49)	(22.09)	(19.50)	(22.56)
Percent of Teens	11.36	16.65	11.82	16.50	21.26	17.04
who are Black	(13.59)	(13.82)	(12.11)	(13.29)	(16.18)	(14.50)
Percent of Teens	12.29	25.73	13.88	25.97	18.70	26.28
who are Hispanic	(15.78)	(22.61)	(16.06)	(22.55)	(18.66)	(23.23)
County-year Observations	63,184	1,210	19,734	1,012	2,002	1,100

Notes: Standard deviations in parentheses. Means and standard deviations weighted by the population of teen women aged 14–19.

2 Robustness Checks

Table S2 reports a series of robustness checks that assess the sensitivity of our preferred estimates to alternative model specification, weighting, and sample criteria. A first set of estimates ("analytic sample") reproduces in tabular form the estimates reported in Fig. 2 in the main text, with these estimates weighted by w_{it}, the number of teen women who were 14–19 in county i and calendar year t. The weighted and unweighted versions of these estimates do not differ in sign and statistical significance, but the weighted estimates are smaller in magnitude than the unweighted estimates.

A next set of estimates examines the robustness of results to alternative sampling criteria. Recall that the estimates reported in the main text excluded counties that received federal funding for abstinence-only sex education to ensure that comparison groups consist of a never-funded condition and a condition in which the first and only source of of county-level federal funding was for more comprehensive sex education. (Additionally, we find that the parallel trends assumption is violated when including these counties.) In this second set of estimates, the point estimates for the CS ATTs are similar in magnitude when weighting (-0.0331 and -0.0333) and not weighting (-0.0437 and -0.0438). However, the standard error for the conditional CS ATT is more than twice as large as the unconditional CS ATT when weighting (0.0221 vs. 0.0107); hence, the conditional CS ATT is not statistically significant when weighting whereas the other three CS ATTs are statistically significant at the .05 level.

In a third set of estimates, the sample consists of counties that exclusively received funding for either abstinence-only or more comprehensive sex education, thus excluding counties that received neither type or both types of funding. These analyses are intended to examine the potential selectivity of the treated counties in these data by comparing them with counties that applied for and received funding for abstinence-only sex education. The treatment condition continues to consist of county-year observations in which a county received federal funding for more comprehensive sex education, but the control condition now consists of: (a) county-year observations in which neither type of funding was received, or (b) county-year observations in which the only source of federal funding was for abstinence-only sex education. Five treated counties had no comparison county in the same state, and were dropped from the sample. The unconditional CS ATTs remain statistically significant and close in magnitude to their row 1 counterparts, but the conditional CS ATTs are somewhat smaller in magnitude and 95% confidence intervals include zero, albeit barely. The unweighted CS ATTs are considerably smaller in magnitude than the weighted CS ATTs, but they remain negative.

A fourth set of estimates drops rural counties from the original sample that excluded counties with abstinence-only funding. This exclusion reduces the number of funded counties from 55 to 46 and the number of counties overall from 2927 to 943. The unweighted and weighted estimated coefficients in Model 1 and 2 are notably smaller than those in the first set of estimates, but the weighted conditional ATT of -0.0328 is nearly identical to our preferred ATT estimate of -0.0329 in row 1.

A fifth set of estimates excludes Colorado and Texas from the sample. In both of these states, access to contraception and abortion changed significantly during the

study period. In Texas, decreases in abortion availability led to increases in teen births (Lindo et al. 2020). In Colorado, funding for contraception at Title X clinics increased and teen births declined as a result (Lindo and Packham 2017). Notably, both of these changes were geographically concentrated in ways that could bias our estimates. The exclusion of counties in these states reduces the total number of counties to 2637 and the number of funded counties to 50, but the estimates remain largely unchanged. As shown below in Fig. S1 estimated effects of funding were large in Colorado, but close to 0 in Texas.

A sixth set of estimates excludes Alaska, Montana, and New Hampshire, three states that had laws requiring parental notification or consent for teens seeking an abortion.

The vast majority of parental notification or consent laws were enacted in the 1990's, with these laws in effect during our study period, but these three states were exceptions. New Hampshire enacted such a law in 2012, Montana's law was active from 2013–2014, and Alaska's law was active from 2010–2016 (Myers and Ladd 2020). Excluding these states reduces the total number of counties to 2843, of which 54 received funding. Previous research has shown that the effect of such laws on abortions varied, with fewer abortions in parts of states that were further from bordering states that did not restrict abortion access (Myers and Ladd 2020). If the geographic distribution of the effects of these laws were to be correlated with the distribution of funding for more comprehensive sex education, this could bias our estimates. The exclusion leads to only minimal changes in our estimates.

A last set of estimates assesses the sensitivity of findings under different model assumptions for the outcome. Recall that our main estimates modeled the outcome as $\log(1 + R)$, where R denotes the county-level teen birth rate. A seventh set of estimates uses an inverse hyperbolic sine transformation of R and yields estimates that differ only slightly from the first set of estimates. An eighth set of estimates models R using a Poisson specification and yields estimates that are somewhat larger than our main set of estimates.

Fig. S1 presents estimates from Model 3 for the 27 states where at least one county received funding, calculated by estimating Model 3 separately within each state. Although there is considerable heterogeneity in the estimated effects by state, 21 out of the 27 point estimates are negative. Fig. S1 thus suggests that no single state or group of states are driving our preferred estimate that funding for more comprehensive sex education led to a 3.3% reduction in county-level teen births.

Table S2: Robustness of estimates to alternative model specifications, weighting, and sample criteria.

	Weighted					Unweighted						
	Coefficient from Model			CS ATT		Coefficient from Model			CS ATT		N Counties	
	1	2	3	Unconditional	Conditional	1	2	3	Unconditional	Conditional	Funded	Total
1. Analytic sample	−0.0812	−0.0781	−0.0447	−0.0360	−0.0329	−0.1178	−0.0945	−0.0540	−0.0395	−0.0352	55	2927
	(0.0162)	(0.0142)	(0.0101)	(0.0131)	(0.0154)	(0.0178)	(0.0196)	(0.0163)	(0.0152)	(0.0180)		
2. Include counties with abstinence-only funding	−0.0791	−0.0750	−0.0243	−0.0331	−0.0333	−0.1154	−0.1022	−0.0542	−0.0437	−0.0438	100	3099
	(0.0122)	(0.0111)	(0.0073)	(0.0107)	(0.0221)	(0.0141)	(0.0150)	(0.0119)	(0.0176)	(0.0115)		
3. Selection on funding	−0.0317	−0.0337	−0.0253	−0.0359	−0.0233	−0.0570	−0.0432	−0.0419	−0.0188	−0.0145	50	141
	(0.0197)	(0.0148)	(0.0125)	(0.0143)	(0.0139)	(0.0188)	(0.0185)	(0.0199)	(0.0188)	(0.0179)		
4. Drop rural counties	−0.0533	−0.0545	−0.0374	−0.0281	−0.0328	−0.0710	−0.0526	−0.0660	−0.0365	−0.0354	46	943
	(0.0169)	(0.0133)	(0.0102)	(0.0130)	(0.0177)	(0.0189)	(0.0210)	(0.0196)	(0.0135)	(0.0176)		
5. Drop Colorado and Texas	−0.0863	−0.0832	−0.0446	−0.0396	−0.0379	−0.1272	−0.1003	−0.0594	−0.0545	−0.0300	50	2637
	(0.0167)	(0.0145)	(0.0103)	(0.0141)	(0.0180)	(0.0185)	(0.0208)	(0.0173)	(0.0163)	(0.0189)		
6. Drop Alaska, Montana, and New Hampshire	−0.0809	−0.0779	−0.0447	−0.0360	−0.0328	−0.1153	−0.0921	−0.0562	−0.0379	−0.0393	54	2843
	(0.0162)	(0.0143)	(0.0101)	(0.0139)	(0.0159)	(0.0177)	(0.0197)	(0.0160)	(0.0160)	(0.0178)		
7. Inverse hyperbolic sine	−0.0830	−0.0802	−0.0474	−0.0373	−0.0395	−0.1201	−0.0953	−0.0559	−0.0406	−0.0401	55	2927
	(0.0180)	(0.0148)	(0.0108)	(0.0143)	(0.0173)	(0.0203)	(0.0217)	(0.0180)	(0.0166)	(0.0202)		
8. Poisson	−0.0494	−0.0585	−0.0361			−0.0998	−0.0979	−0.0527			55	2927
	(0.0304)	(0.0155)	(0.0098)			(0.0172)	(0.0161)	(0.0108)				

Notes: Standard errors in parentheses. See text for additional details.

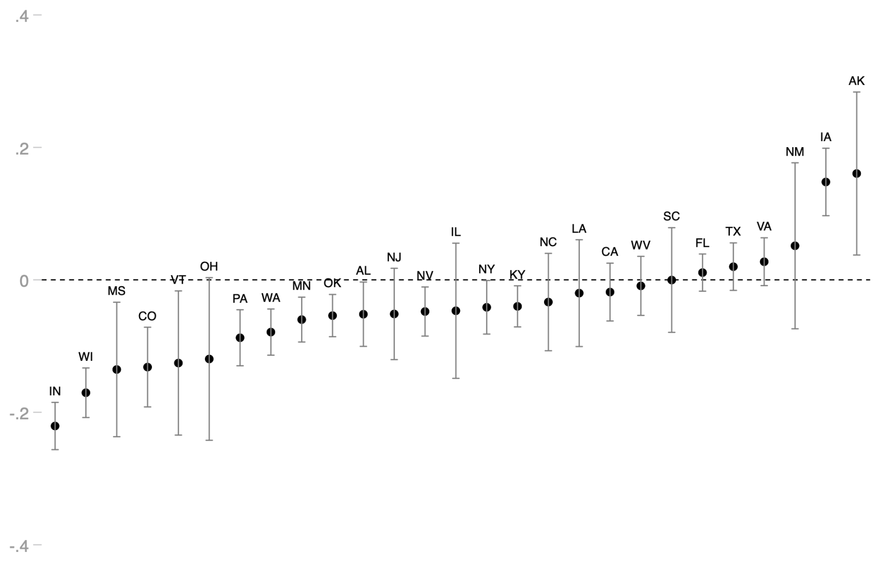

Figure S1: Estimated causal effects by state.
Notes: 95% confidence intervals. Results from Model 3 estimated separately by state.

3 Operationalizing treatment at the state level.

In our review of the prior studies, we noted that Fox et al. (2019) was the only other published study to adopt a difference-in-differences design in order to obtain credibly causal estimates of more comprehensive sex education on teen births. But we also noted potential issues with their state-level difference-in-differences design and, in particular, that because federal funding under the Teen Pregnancy Prevention (TPP) program allocated funding to sub-state entities, this raised the possibility of measurement error in their state-level treatment variable. The analyses in this section thus proceed, as in Fox et al. by operationalizing treatment at the state level. We use a binary variable equal to one if *any* county i in the state j received TPP funding in year t.

The results are presented below in Figure S2. The estimates from Models 1 and 2 and the unconditional CS ATT are similar to those at the county-level. But when the models are adjusted for state-level trends, either by including those trends via state-specific linear trend terms in Model 3 or by estimating

the conditional CS ATTs, the estimated effects of treatment are close to zero.

These results thus illustrate the empirical consequences of our county-level specification as opposed to a state-level one.

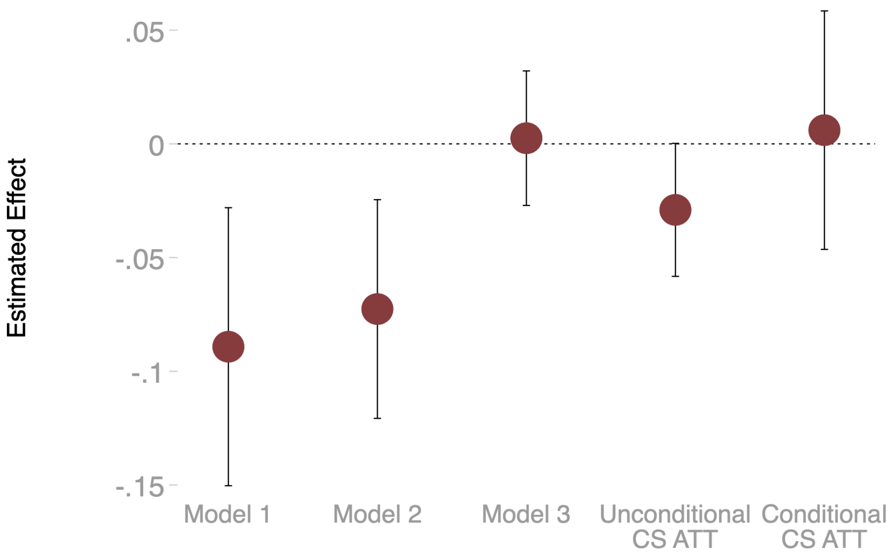

Figure S2: Estimated effects when funding is allocated at the state level.
Notes: 95% confidence intervals.

References

1. Lindo, J. M., Myers, C. K., Schlosser, A., & Cunningham, S. (2020). How Far Is Too Far? New Evidence on Abortion Clinic Closures, Access, and Abortions. Journal of Human Resources, 55(4), 1137–1160. https://doi.org/10.3368/jhr.55.4.1217-9254R3

2. Lindo, J. M., & Packham, A. (2017). How much can expanding access to long-acting reversible contraceptives reduce teen birth rates? American Economic Journal: Economic Policy, 9(3), 348–376. https://doi.org/10.1257/pol.20160039

3. Myers, C., & Ladd, D. (2020). Did parental involvement laws grow teeth? The effects of state restrictions on minors' access to abortion. Journal of Health Economics, 71, 102302. https://doi.org/10.1016/j.jhealeco.2020.102302

4. Fox, A. M., Himmelstein, G., Khalid, H., & Howell, E. A. (2019). Funding for Abstinence-Only Education and Adolescent Pregnancy Prevention: Does State Ideology Affect Outcomes? American Journal of Public Health, 109(3), 497–504. https://doi.org/10.2105/AJPH.2018.304896
